# BK and JC polyomaviruses and risk of urothelial bladder carcinoma: a preliminary study in the northern shores of Persian Gulf, Iran

**DOI:** 10.1186/s13027-022-00463-x

**Published:** 2022-09-19

**Authors:** Reza Taherkhani, Fatemeh Farshadpour

**Affiliations:** Department of Virology, School of Medicine, Bushehr University of Medical Sciences, Moallem Street, Bushehr, 751463334 Iran

**Keywords:** Urothelial bladder carcinoma, Human polyomavirus, BK polyomavirus, John Cunningham polyomavirus, Prevalence, Genotype, Risk factors, Iran

## Abstract

**Background:**

Bladder cancer is a challenging public health concern in South of Iran because of its high prevalence and the related medical expenses. Although the exact etiology of bladder cancer remains unknown, given the cell transforming ability and oncogenic potential of the members of *Polyomaviridae* families, this study was conducted to evaluate the magnitude of BK polyomavirus (BKPyV) and John Cunningham polyomavirus (JCPyV) among patients with bladder cancer residents in the northern shores of the Persian Gulf, South of Iran.

**Methods:**

Totally 211 patients with bladder cancer were enrolled in this study. Bladder biopsy samples of these patients and patients with interstitial cystitis as well as autoptic samples of healthy bladder were tested for detection of BKPyV and JCPyV by semi-nested PCR–RFLP followed by sequencing.

**Results:**

BKPyV and JCPyV were detected in 1.7% and 6.1% of bladder cancer samples, respectively. These samples were infected with JCPyV genotypes 2, 3 and 6 and BKPyV genotypes I and IV. BKPyV and JCPyV coinfection was detected in 2 samples. Moreover, one of the healthy bladder samples was positive for BKPyV, and one of the interstitial cystitis samples was positive for JCPyV. Although the majority of infected patients were in the age group 70–79 years, male, residents in Tangestan, stage Ta–T1, and low-grade and high-grade papillary urothelial carcinoma, the prevalence of BKPyV and JCPyV among patients with bladder cancer was not statistically associated with age, gender, place of residency, and stage and grade of the tumor.

**Conclusion:**

Despite identifying BKPyV and JCPyV in a number of bladder cancer biopsy specimens and the high prevalence of bladder cancer among people resident in South of Iran, it is suggested that these viruses are unlikely to be effective causative factors in bladder carcinogenesis in this region. Therefore, environmental risk factors and genetic backgrounds may have a more prominent role than human polyomaviruses in the development of bladder cancer in South of Iran.

## Introduction

Bladder cancer, with approximately 570,000 new cases and an estimated 210,000 deaths annually, is considered to be the tenth most common cancer worldwide [1]. Over 90% of bladder cancers are transitional cell carcinoma, which are currently classified as urothelial bladder carcinoma, whereas 5% are squamous cell carcinoma and 2% are adenocarcinoma [1]. Cigarette smoking, prolonged use of antineoplastic drugs, arsenic in drinking water, occupational exposure to polycyclic hydrocarbons, aromatic amines and ionizing radiation, genetic background, chronic cystitis, schistosomiasis, and some bacterial and viral infections are possible risk factors for bladder cancer [2, 3]. Viral agents are responsible for 15–20% of all human cancers [2]. Among cancer-related viruses, BK polyomavirus (BKPyV) and John Cunningham polyomavirus (JCPyV) have been suggested as risk factors for the development of bladder cancer. This suggestion has been strengthened by the cell transforming ability and the oncogenic potential of some members of the *Polyomaviridae* family, including Merkel cell polyomavirus (MCPyV), SV40, Trichodysplasia spinulosa polyomavirus (TSPyV), JCPyV, BKPyV, HPyV6 and HPyV7 [4–6].

Human polyomaviruses are small, icosahedral, non-enveloped viruses in the family *Polyomaviridae*. Their circular double-stranded DNA genome contains an early coding region, which encodes two regulatory non-structural proteins (large and small tumor antigens), a late coding region, which encodes three structural proteins (VP1, VP2 and VP3) and a non-structural agnoprotein, and a non-coding region in between that regulates replication and transcription [7, 8]. These viruses might be transmitted through exposure to contaminated food, water and surfaces or via person-to-person contact, respiratory tract and organ transplantation [4]. Most people are exposed to these viruses in early childhood. Primary infection with these viruses during childhood results in life-long latent infection in peripheral-blood leukocytes, kidneys, urinary tract, lymphoid tissue, bone marrow and brain [7, 8]. Although the primary infection in immunocompetent individuals is usually asymptomatic or mild, in immunocompromised patients, infection with JCPyV is capable of causing progressive multifocal leukoencephalopathy, while infection with BKPyV can progress to hemorrhagic cystitis and nephropathies [4]. Moreover, the large tumor antigen (LTAg) of BKPyV, JCPyV and SV40 might induce cell proliferation and downregulation of apoptosis by interacting with pRb and p53 tumor suppressor proteins, leading to cell transformation and tumorigenesis [5, 7].

Bladder cancer is a challenging public health concern in the South of Iran because of its high prevalence in this region and the related medical expenses. Age-standardized incidence rate (ASR) of bladder cancer is 19.00 per 100,000 in men and 6.83 per 100,000 in women in Bushehr province, South of Iran [9]. Although the exact etiology of bladder cancer remains unknown, given the cell transforming ability and the oncogenic potential of some members of *Polyomaviridae* families such as MCPyV, SV40, TSPyV, JCPyV and BKPyV, this study was conducted to evaluate the magnitude of BKPyV and JCPyV among patients with bladder cancer resident in the northern shores of the Persian Gulf, the South of Iran. In addition, the possible association between these infectious pathogens and the stage and grade of bladder cancer was evaluated. This is the first study on the molecular epidemiology of cancer-related viruses in this region. Information regarding the possible role of these viral agents in the development of bladder cancer might affect prevention and treatment strategies to reduce the incidence of bladder cancer.

## Subjects and methods

### Patients and sample collection

All of the patients with bladder cancer referred to the hospitals of Bushehr University of Medical Sciences located in southern Iran were included consecutively in this study. The patients were excluded from entering the study if they had a history of other malignancies or exhibited evidence of cancer metastasized to bladder tissue from another organ. Formalin-fixed paraffin-embedded bladder biopsy samples of 211 patients with bladder cancer and 11 patients with interstitial cystitis (non-cancerous) were collected from the archives of the pathology department of the hospitals. In addition, fresh frozen autoptic samples of healthy bladder (non-neoplastic and non-cystitis) were taken from 19 deceased persons who died due to car accidents. All patients and the legal guardians of the deceased persons gave written informed consent to use their samples for viral detection and analysis. Grading and staging of tumors were carried out according to the World Health Organization/International Society of Urological Pathology (WHO/ISUP) and the American Joint Committee on Cancer/tumor, nodes, metastases (AJCC/TNM) staging system, respectively [1, 10, 11]. Demographic data and clinicopathologic characteristics were obtained from the medical record of the patients at the pathology department of the hospitals. This descriptive-analytical study was conducted with the approval of the Ethical Committee of the Bushehr University of Medical Sciences (Research project number: B-93-16-18) and funded by the Deputy Research and Affairs of the University (Grant Number: 8038). Besides, all methods were performed in accordance with the relevant guidelines and regulations.

### DNA extraction

Ten-micrometer-thick tissue Sections (10 sections) were deparaffinized with 1 ml xylene (2 times), then wash with absolute ethanol (3 times) and 70% ethanol (1 time). DNA was extracted from the deparaffinized tissue samples and fresh frozen tissue samples using the High Pure PCR Template Preparation Kit (Roche, Mannheim, Germany) according to the manufacturer's instructions. The quantity and purity of the extracted DNA were evaluated by a NanoDrop 1000 Spectrophotometer (Thermo Scientific, Waltham, Mass., USA), then the extracted DNA was aliquoted and stored at − 70 °C until use. In order to confirm DNA integrity and to avoid false-negative results due to inappropriate DNA extraction or the presence of PCR inhibitors, a PCR assay was performed for all samples using specific primers for the human β-globin gene or the KRAS gene as the internal control [12, 13].

### PCR amplification and sequencing

The detection of BKPyV and JCPyV was performed by semi-nested PCR-restriction fragment length polymorphism (PCR–RFLP) method, targeting the LTAg region of the genome. The 276 bp length fragment from the LTAg region was amplified in the first round PCR using outer primers AAGTCTTTAGGGTCTTCTACC (PEP-1F) and CAGRGATCTAAAGCTTTAAGG (DP-R1). The second-round PCR was performed using inner primers AAGTCTTTAGGGTCTTCTACC (PEP-1F) and GGTGCCAACCTATGGAACAGA (PEP-R2) [14]. The amplified 176 bp length fragments from the second-round PCR were digested by BamHI restriction endonuclease to distinguish between BKPyV and JCPyV. In the case of JCPyV, the PCR amplicons were cleaved into 122 bp and 54 bp length fragments by BamHI, whereas in the case of BKPyV, no digestion occurred and the amplicons remained intact. The viral identity or the results of PCR–RFLP were also confirmed by sequencing. Moreover, the extracted DNA of positive samples was amplified by semi-nested PCR assay, which amplifies the VP1 regions of the BKPyV and JCPyV genome. The 562 bp and 433 bp length fragments from the VP1 region were amplified using outer primers [TGTACGGGACTGTAACACC (BKJC-f) and TCTGGGTACTTTGTYCTGTA (PoE2as)] and inner primers [GGAGGAGTAGAAGTTCTAGAA (PoE1s) and TCTGGGTACTTTGTYCTGTA (PoE2as)], respectively [15]. The amplified 433 bp length fragment was sequenced by Sanger dideoxy sequencing technology to determine the genotypes of BKPyV and JCPyV (Macrogen Co., Korea). Known BKPyV and JCPyV positive clinical samples and PCR-grade water with PCR reagents were used as the positive and negative controls of the PCR assay, respectively. The sequences of primers, PCR conditions, and regions in the genome for detection of BKPyV and JCPyV are summarized in Table 1.

**Table 1:** Sequences of primers for detection of BK polyomavirus (BKPyV), John Cunningham polyomavirus (JCPyV) and internal controls

Virus	Primers name	Sequences of primers 5′ → 3′	Gene	Region in genome	Annealing temperature (°C)	Size	References
BKPyV and JCPyV	PEP-1F	AAGTCTTTAGGGTCTTCTACC	Large T antigen	4497–4518	56	276 bp	[14]
	DP-R1	CAGRGATCTAAAGCTTTAAGG		4764–4743			
	PEP-1F	AAGTCTTTAGGGTCTTCTACC		4497–4518	56	176 bp	
	PEP-R2	GGTGCCAACCTATGGAACAGA		4673–4652			
BKPyV and JCPyV	BKJC-f	TGTACGGGACTGTAACACC	VP1	1629–1648	57	562 bp	[15]
	PoE2as	TCTGGGTACTTTGTYCTGTA		2191–2171			
	PoE1s	GGAGGAGTAGAAGTTCTAGAA		1758–1779	55	433 bp	
	PoE2as	TCTGGGTACTTTGTYCTGTA		2191–2171			
Inernal controls	PCO3F	ACACAACTGTGTTCACTAGC	β globin	5,248,179	55	110 bp	[12]
	PCO4R	CAACTTCATCCACGTTCACC		5,248,288			
	KRAS-F	GGTGAGTTTGTATTAAAAGGTACTGG	KRAS	10,423	55	263 bp	[13]
	KRAS-R	TCCTGCACCAGTAATATGCA		10,666			

### Phylogenetic analysis

The isolated sequences were aligned and compared with the reference sequences representing the standard genotypes of BKPyV and JCPyV available at the nucleotide database of the NCBI by the ClustalW program in the MEGA software version 7.0 (Biodesign Institute, Tempe, AZ, USA). Then, the phylogenetic trees were constructed by the neighbor-joining methods.

### Statistical analysis

Statistical analyses were performed using SPSS 17 package program (SPSS Inc., Chicago, IL, USA). Data were presented as mean ± standard deviation (SD), percentages, and frequencies. The Student's t-test and the chi-square test or the Fisher exact test were used for data analysis, and P values < 0.05 were defined statistically significant.

## Results

Information of 211 patients with bladder cancer was available at the archives of the pathology department of the hospitals located in southern Iran. Of these, 74 patients were from Bushehr, 34 patients were from Tangestan, 44 patients were from Dashtestan, 27 patients were from Dashti, 13 patients were from Dayer, 3 patients were from Deylam, 5 patients were from Kangan, 10 patients were from Genaveh, and 1 patient was from Jam city. The mean age ± SD of patients with bladder cancer was 64.52 ± 13.78 years with a range of 27–92 years. The majority of patients were in the age group 50–69 years (48.34%), male (76.8%), residents in Bushehr city (35.1%), and had low-grade papillary urothelial carcinoma (39.8%) and were in stage Ta–T1 (89.1%) (Table 2). Patients in stage T2–T3 (71.57 ± 13.15) had a higher mean age compared to patients in stage Ta–T1 (63.23 ± 13.94). Moreover, patients with high-grade papillary urothelial carcinoma (71.1 ± 11.68) had a higher mean age compared to patients with urothelial papilloma (50.0 ± 7.26). Overall, the mean age of patients increased with increasing the stage and grade of cancer.

**Table 2:** Demographic data and tumor characteristics of patients with bladder cancer in the South of Iran

	No. of all patients (%): 211 (100%)	No. of formalin-fixed paraffin-embedded bladder cancer biopsy samples (%): 181 (100%)
*Age groups (years)*		
27–39	9 (4.3)	7 (3.9)
40–49	21 (10.0)	15 (8.3)
50–59	50 (23.7)	46 (25.4)
60–69	52 (24.6)	45 (24.9)
70–79	46 (21.8)	39 (21.5)
≥ 80	33 (15.6)	29 (16.0)
*Gender*		
Male	162 (76.8)	138 (76.2)
Female	49 (23.2)	43 (23.8)
*Place of residence (city)*		
Bushehr	74 (35.1)	63 (34.8)
Tangestan	34 (16.1)	27 (14.9)
Dashtestan	44 (20.9)	38 (21.0)
Dashti	27 (12.8)	25 (13.8)
Dayer	13 (6.2)	11 (6.1)
Deylam	3 (1.4)	3 (1.7)
Kangan	5 (2.4)	5 (2.8)
Genaveh	10 (4.7)	8 (4.4)
Jam	1 (0.5)	1 (0.6)
*Stage of tumor*		
Ta–T1	188 (89.1)	166 (91.7)
T2–T3	23 (10.9)	15 (8.3)
*Grade of tumor*		
Urothelial papilloma	4 (1.9)	4 (2.2)
Papillary urothelial neoplasms of low malignant	66 (31.3)	62 (34.3)
Low-grade papillary urothelial carcinoma	84 (39.8)	68 (37.6)
High-grade papillary urothelial carcinoma	57 (27.0)	47 (26.0)

**Fig. 1:**
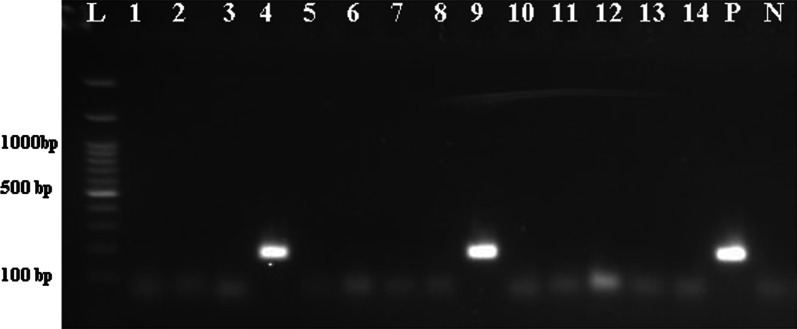
Electrophoresis of PCR products of LTAg region of BK polyomavirus (BKPyV) and John Cunningham Polyomavirus (JCPyV) genome extracted from bladder tissue samples on 2% agarose gel. L, 100-bp DNA ladder; N, negative control; P, positive control; 4 and 9, amplified products (~ 176 bp)

**Fig. 2:**
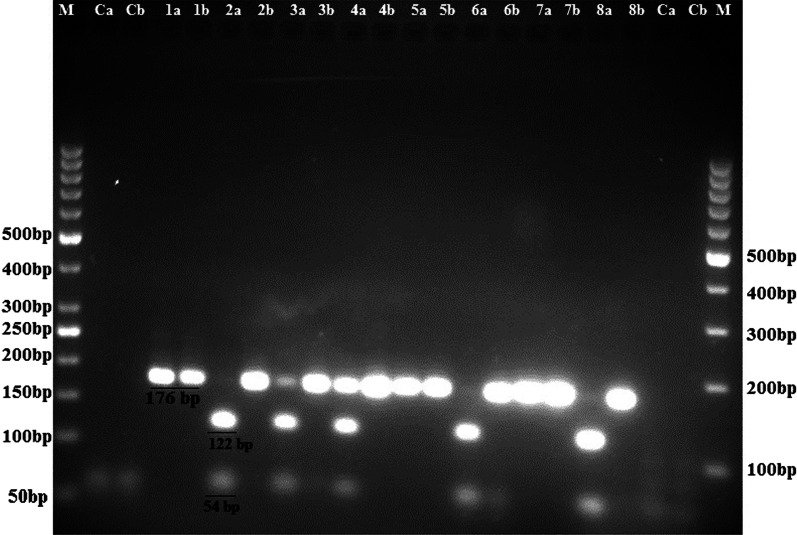
Electrophoresis of PCR–RFLP products of LTAg region of human polyomaviruses genome extracted from bladder tissue samples on 2% agarose gel to distinguish between BKPyV and JCPyV. M, 50-bp DNA ladder; Ca and Cb, Negative controls; 1a, 2a, 3a, 4a, 5a, 6a, 7a and 8a, PCR products after BamHI endonuclease digestion; 1b, 2b, 3b, 4b, 5b, 6b, 7b and 8b, PCR products before digestion; 1a, 3a, 4a, 5a, 7a, Undigested PCR amplicons of BKPyV by BamHI restriction endonuclease (176 bp length fragments); 2a, 3a, 4a, 6a, 8a, Digested PCR amplicons of JCPyV by BamHI restriction endonuclease (122 bp and 54 bp length fragments); 3a and 4a, BKPyV and JCPyV coinfection; Ca and Cb, Negative controls; M, 100-bp DNA ladder

**Fig. 3:**
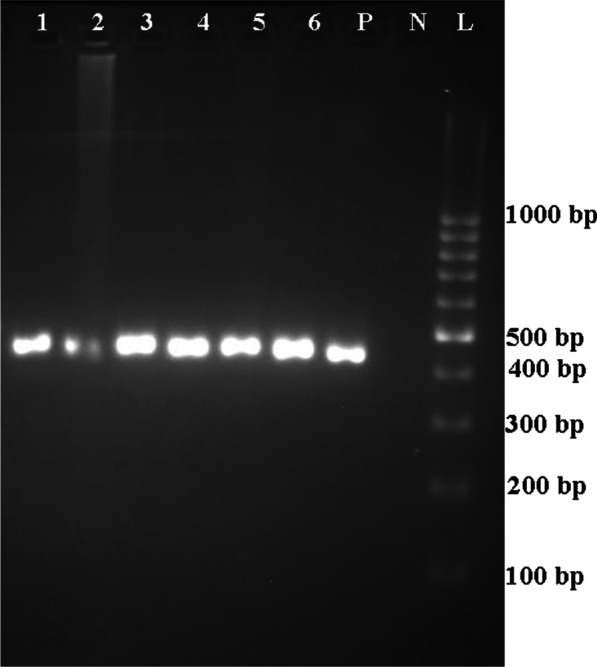
Electrophoresis of PCR products of VP1 region of BK polyomavirus (BKPyV) and John Cunningham polyomavirus (JCPyV) genome extracted from bladder tissue samples on 2% agarose gel. L, 100-bp DNA ladder; N, negative control; P, positive control; 1, 2, 3, 4, 5 and 6, amplified products (~ 433 bp)

**Fig. 4:**
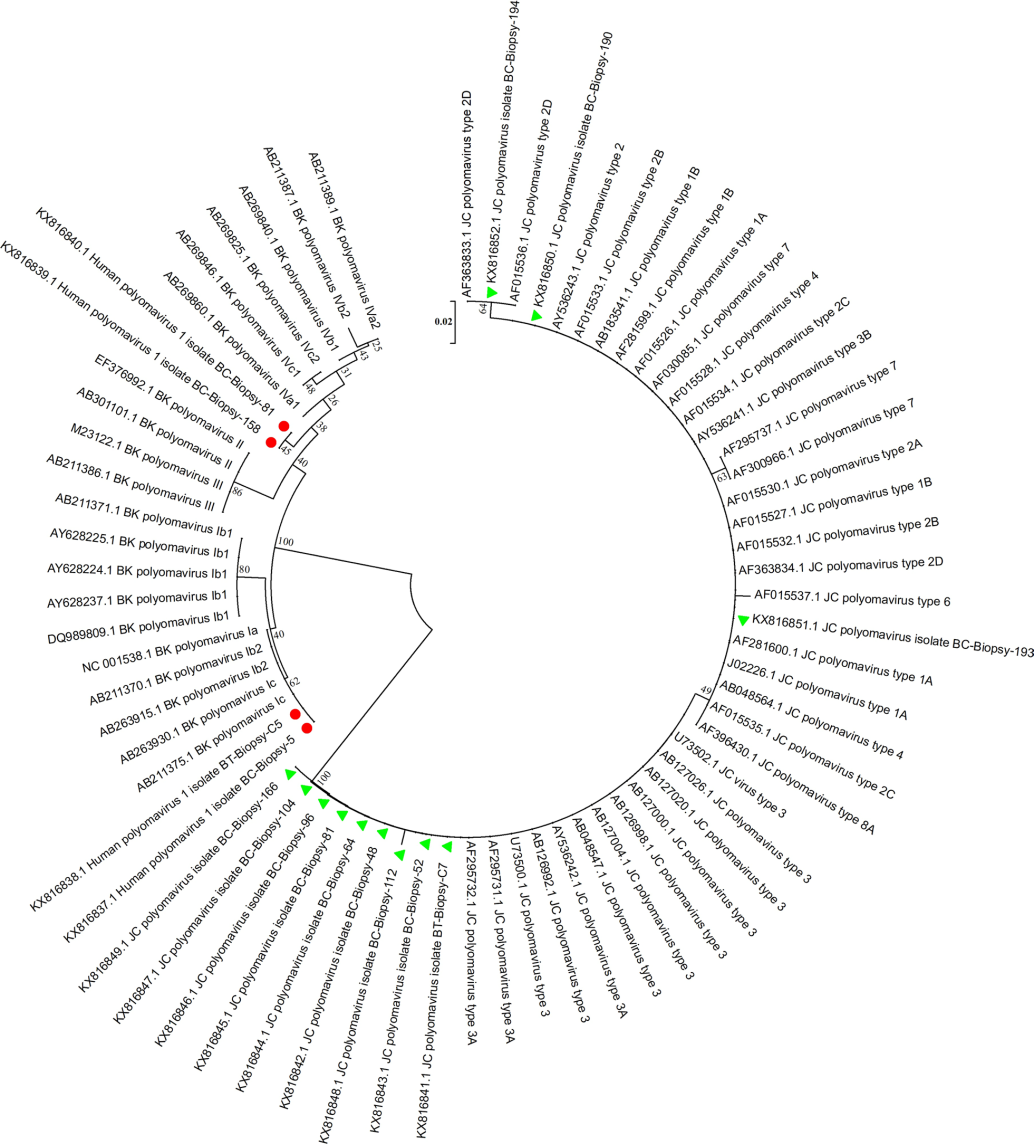
Neighbor-joining phylogenetic tree based on ~ 176 bp nucleotide sequence of the LTAg region of BKPyV (red mark) and JCPyV (green mark) isolates from the bladder tissue samples of patients in South of Iran. Bootstrap re-sampling strategy and reconstruction were carried out 1000 times to confirm the reliability of the phylogenetic tree

**Fig. 5:**
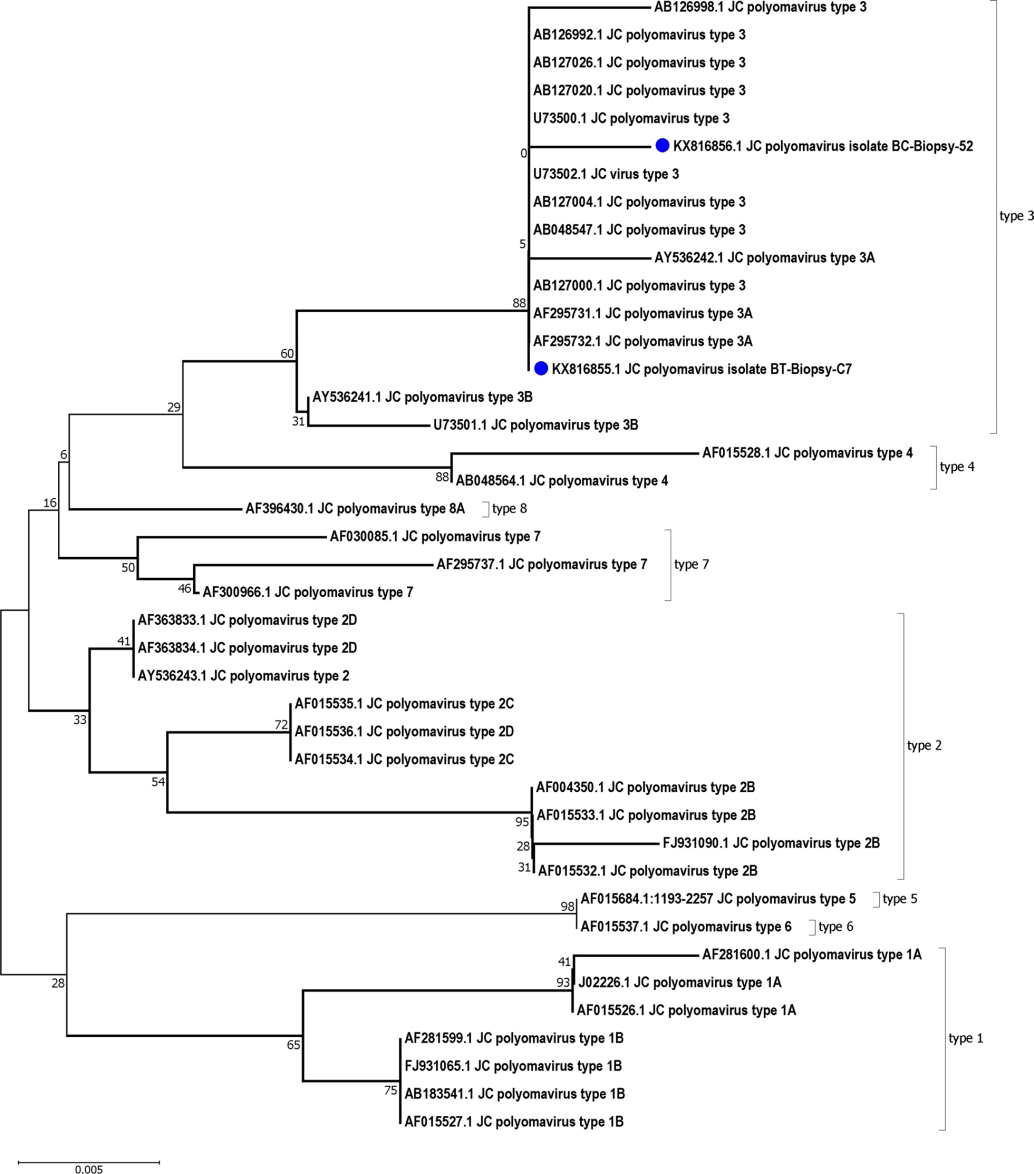
Neighbor-joining phylogenetic tree based on ~ 433 bp nucleotide sequence of the VP1 region of JCPyV isolates from the bladder tissue samples of patients in the South of Iran (blue mark). Bootstrap re-sampling strategy and reconstruction were carried out 1000 times to confirm the reliability of the phylogenetic tree

**Fig. 6:**
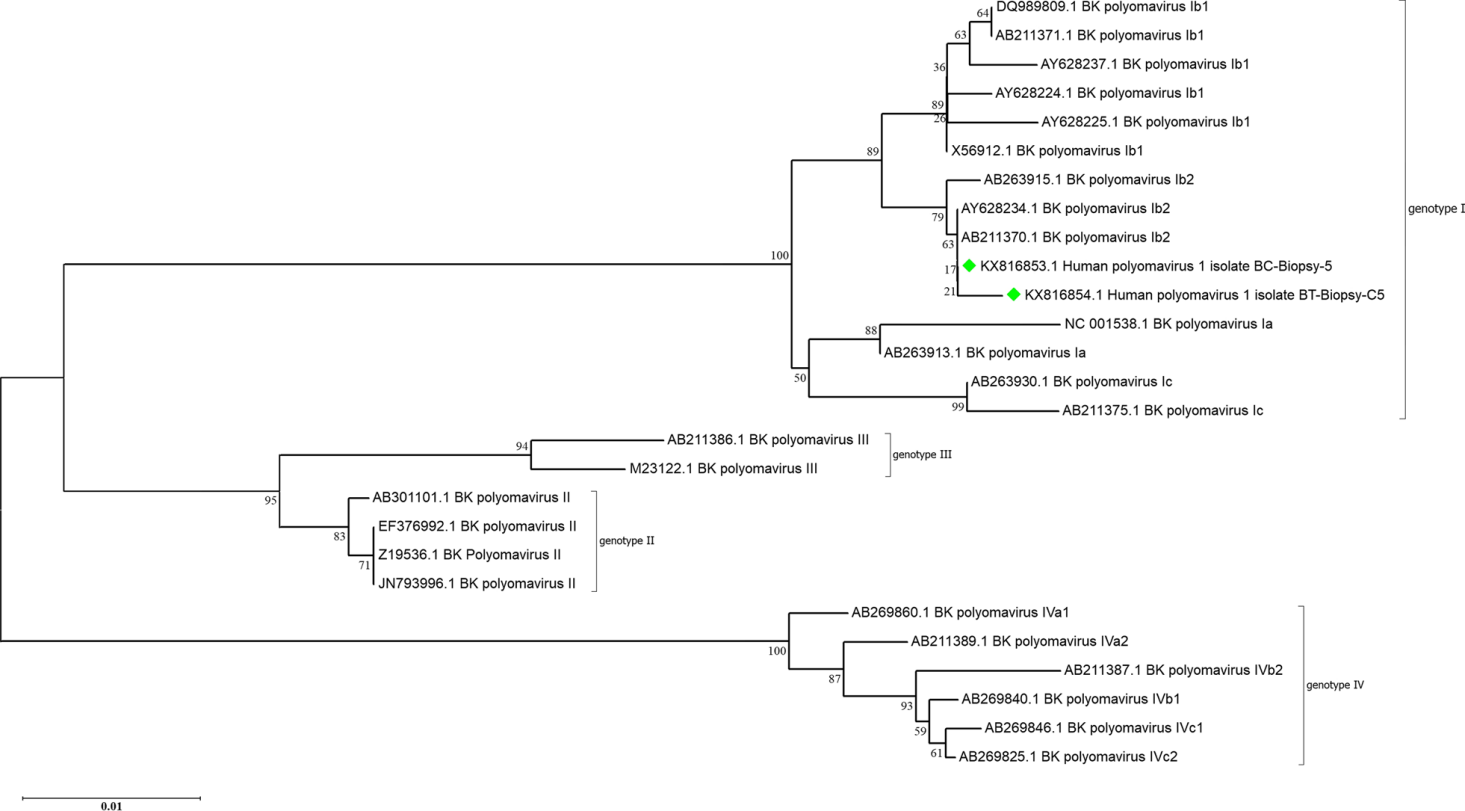
Neighbor-joining phylogenetic tree based on ~ 433 bp nucleotide sequence of the VP1 region of BKPyV isolates from the bladder tissue samples of patients in South of Iran (green mark). Bootstrap re-sampling strategy and reconstruction were carried out 1000 times to confirm the reliability of the phylogenetic tree

Of 211 patients with bladder cancer, 30 patients were excluded due to the absence of formalin-fixed paraffin-embedded bladder biopsy samples or poor tissue quality following DNA extraction. Overall, 181 cancerous bladder samples and 30 non-cancerous bladder samples were tested for detection of BKPyV and JCPyV. These 30 non-cancerous samples included 11 interstitial cystitis and 19 healthy bladder samples, and were taken from 16 men and 14 women, ages ranging from 22 to 84 years (58.0 ± 18.78). Of these, 18 samples were from Bushehr city, 5 samples were from Dashtestan, 2 samples were from Genaveh, 3 samples were from Tangestan and 2 samples were from Dashti. BKPyV and JCPyV were detected in 1.7% (3/181) and 6.1% (11/181) of the bladder cancer samples, respectively. One of the healthy bladder samples was positive for BKPyV, and one of the interstitial cystitis samples was positive for JCPyV (Figs. 1, 2 and 3). These samples were infected with JCPyV genotypes 2 (two samples), 3 (nine samples) and 6 (one sample) and BKPyV genotypes I (two samples) and IV (two samples) (Figs. 4, 5 and 6). The internal control was positive in all samples.

The BKPyV positive patients were in the age group 70 to ≥ 80 years, male, residents in Tangestan and Dashti and had papillary urothelial neoplasms of low malignant, low-grade papillary urothelial carcinoma and high-grade papillary urothelial carcinoma in the stage Ta–T1 (Table 3). The majority of JCPyV positive patients were in the age group 70–79 years (54.5%), male (81.8%), residents in Tangestan and Bushehr (54.6%), the stage Ta–T1 (90.9%), and had low-grade and high-grade papillary urothelial carcinoma (72.8%) (Table 4). Moreover, BKPyV and JCPyV coinfection was detected in 2 samples. Although the rate of BKPyV and JCPyV positivity varied by age distribution, gender, place of residency, stage and grade of tumor, the prevalence of BKPyV and JCPyV among patients with bladder cancer was not statistically associated with age, gender, place of residency, and stage and grade of the tumor (*P*-value > 0.05) (Tables 3 and 4).

**Table 3:** Prevalence of BK polyomavirus (BKPyV) according to socio-demographic data and tumor characteristics among patients with bladder cancer in the South of Iran

	No. of all participants (%): 181 (100%)	No. of BKPyV positive subjects (%): 3 (1.7%)	No. of BKPyV negative subjects (%):178 (98.3%)	*P*-Value
*Age groups (years)*				0.374
27–39	7 (3.9)	0 (0.0)	7 (3.9)	
40–49	15 (8.3)	0 (0.0)	15 (8.4)	
50–59	46 (25.4)	0 (0.0)	46 (25.8)	
60–69	45 (24.9)	0 (0.0)	45 (25.3)	
70–79	39 (21.5)	2 (66.7)	37 (20.8)	
≥ 80	29 (16.0)	1 (33.3)	28 (15.7)	
*Gender*				0.330
Male	138 (76.2)	3 (100.0)	135 (75.8)	
Female	43 (23.8)	0 (0.0)	43 (24.2)	
*Place of residence (city)*				0.387
Bushehr	63 (34.8)	0 (0.0)	63 (35.4)	
Tangestan	27 (14.9)	2 (66.7)	25 (14.0)	
Dashtestan	38 (21.0)	0 (0.0)	38 (21.3)	
Dashti	25 (13.8)	1 (33.3)	24 (13.5)	
Dayer	11 (6.1)	0 (0.0)	11 (6.2)	
Deylam	3 (1.7)	0 (0.0)	3 (1.7)	
Kangan	5 (2.8)	0 (0.0)	5 (2.8)	
Genaveh	8 (4.4)	0 (0.0)	8 (4.5)	
Jam	1 (0.6)	0 (0.0)	1 (0.6)	
*Stage of tumor*				0.600
Ta–T1	166 (91.7)	3 (100.0)	163 (91.6)	
T2–T3	15 (8.3)	0 (0.0)	15 (8.4)	
*Grade of tumor*				0.986
Urothelial papilloma	4 (2.2)	0 (0.0)	4 (2.2)	
Papillary urothelial neoplasms of low malignant	62 (34.3)	1 (33.3)	61 (34.3)	
Low-grade papillary urothelial carcinoma	68 (37.6)	1 (33.3)	67 (37.6)	
High-grade papillary urothelial carcinoma	47 (26.0)	1 (33.3)	46 (25.8)

**Table 4:** Prevalence of John Cunningham polyomavirus (JCPyV) according to socio-demographic data and tumor characteristics among patients with bladder cancer in the South of Iran

	No. of all participants (%): 181 (100%)	No. of JCPyV positive subjects (%): 11 (6.1%)	No. of JCPyV negative subjects (%):170 (93.9%)	Adjusted OR (95% CI)	*P*-Value
*Age groups (years)*					
27–39	7 (3.9)	2 (18.2)	5 (2.9)	1.0	
40–49	15 (8.3)	1 (9.1)	14 (8.2)	5.600 (0.412–76.049)	0.196
50–59	46 (25.4)	0 (0.0)	46 (27.1)	0.000	0.997
60–69	45 (24.9)	2 (18.2)	43 (25.3)	8.600 (0.984–75.151)	0.052
70–79	39 (21.5)	6 (54.5)	33 (19.4)	2.200 (0.344–14.079)	0.405
≥ 80	29 (16.0)	0 (0.0)	29 (17.1)	0.000	0.998
*Gender*					
Male	138 (76.2)	9 (81.8)	129 (75.9)	1.0	
Female	43 (23.8)	2 (18.2)	41 (24.1)	1.430 (0.297–6.888)	0.655
*Place of residence (city)*					
Bushehr	63 (34.8)	2 (18.2)	61 (35.9)	1.0	
Tangestan	27 (14.9)	4 (36.4)	23 (13.5)	0.189 (0.032–1.100)	0.064
Dashtestan	38 (21.0)	1 (9.1)	37 (21.8)	1.213 (0.106–13.848)	0.876
Dashti	25 (13.8)	1 (9.1)	24 (14.1)	0.787 (0.068–9.087)	0.848
Dayer	11 (6.1)	1 (9.1)	10 (5.9)	0.328 (0.027–3.962)	0.380
Deylam	3 (1.7)	1 (9.1)	2 (1.2)	0.066 (0.004–1.06)	0.055
Kangan	5 (2.8)	0 (0.0)	5 (2.9)	0.000	0.999
Genaveh	8 (4.4)	1 (9.1)	7 (4.1)	0.230 (0.018–2.866)	0.253
Jam	1 (0.6)	0 (0.0)	1 (0.6)	0.000	1.000
*Stage of tumor*					
Ta–T1	166 (91.7)	10 (90.9)	156 (91.8)	1.0	
T2–T3	15 (8.3)	1 (9.1)	14 (8.2)	0.897 (0.107–7.530)	0.921
*Grade of tumor*					
Urothelial papilloma	4 (2.2)	0 (0.0)	4 (2.4)	1.0	
Papillary urothelial neoplasms of low malignant	62 (34.3)	3 (27.3)	59 (34.7)	0.000	0.999
Low-grade papillary urothelial carcinoma	68 (37.6)	4 (36.4)	64 (37.6)	0.000	0.999
High-grade papillary urothelial carcinoma	47 (26.0)	4 (36.4)	43 (25.3)	0.000	0.999

## Discussion

Bladder cancer is the 9th leading cause of cancer-related deaths worldwide, causing nearly 210.000 deaths each year [1]. Among possible risk factors for bladder cancer, infectious pathogens are of particular interest for their carcinogenesis properties. Since infectious agent-related cancers can be predictable and preventable by screening and vaccination programs [16]. However, to achieve this goal, the role of infectious pathogens in the development of specific cancers should be confirmed. Therefore, this study was performed to detect BKPyV and JCPyV in bladder cancer tissues and to screening for the presence of these viral pathogens among patients with bladder cancer. In this study, BKPyV and JCPyV were detected in 1.7% and 6.1% of the bladder cancer biopsy specimens, respectively.

The association between human polyomaviruses and bladder carcinoma continues to be controversial. While some studies suggested an association between BKPyV and JCPyV infections and bladder carcinoma [17–19], the other studies denied this association [3, 20–22]. These studies have been performed in different populations with different environmental risk factors and genetic backgrounds and have failed to reach a consensus. A recent study proposed the role of BKPyV in the development of bladder cancer via the anti-viral apolipoprotein B mRNA editing enzyme catalytic polypeptide (APOBEC)-mediated damage of the urothelial genome. In this hit-and-run mode of carcinogenesis, despite inducing APOBEC3B expression by LTAg of BKPyV, the causative viral agent is absent in the tumoral tissue probably due to immune clearance of BKPyV [23]. The present study, despite identifying BKPyV and JCPyV in a number of bladder cancer biopsy specimens in the South of Iran, suggests that these viruses are unlikely to be effective causative factors in bladder cancer in this region. Nevertheless, prospective cohort studies are needed to reach a more definite conclusion and to achieve further understanding of the association between BKPyV and JCPyV infections and the development of bladder cancer in an Iranian population.

The prevalence of 1.7% for BKPyV observed in this study is higher than that reported among patients with bladder cancer in Hungary (0.0%) [22] but lower than those reported in the United Kingdom (3.33%) [3], the United States (5.5%) [21], Mashhad (North-East of Iran) (13.7%) [24] and Italy (55%) [17]. Moreover, the prevalence of 6.1% for JCPyV observed in this study is higher than that reported among patients with bladder cancer in the United Kingdom (0.87%) [3] but lower than that reported in Italy (25%) [17]. These variations in the prevalence of human polyomaviruses in these studies could be due to differences in the type of specimen, processing methods, the sensitivity of the detection assays, and sociodemographic characteristics of the study population.

In this study, multiple samples from different parts of the tumoral tissue were tested for each patient. Since BKPyV and JCPyV may not infect all cancerous parts equally, testing the samples outside the infected site can lead to false-negative results. In addition, a nested PCR method was used to detect BKPyV and JCPyV in bladder tissue specimens. Due to the low number of copies of viral DNA in tissue specimens, a nested PCR assay is more useful for the detection of BKPyV and JCPyV than conventional PCR assays. So that all positive samples were identified in the second round of PCR. However, the risk of contamination, which leads to false-positive results, is higher in nested PCR assay than in one-step PCR assay [25]. In this study, to avoid contamination during PCR procedures, strict quality controls were applied. Moreover, positive and negative controls were used to ensure the accuracy of the results. Accidental contamination at the time of sample processing, which can lead to false-positive results, is another issue. During sectioning each sample, the necessary precautions were taken to avoid cross-contamination with the next sample.

In this study, the prevalence of BKPyV and JCPyV among patients with bladder cancer was not statistically associated with age, gender, place of residency, and the stage and grade of bladder cancer, although the majority of infected patients were in the age group 70–79 years, male, residents in Tangestan, stage Ta-T1, and low-grade and high-grade papillary urothelial carcinoma. Similarly, a study from the United Kingdom reported no association between the prevalence of BKPyV and JCPyV and stage and grade of the tumor [3]. Bladder cancer commonly affects older ages, with a peak incidence in the 7th and 8th decades of life, and is more common in men [17]. In this study, the mean age of patients increased with increasing the stage and grade of bladder cancer. So that patients in stage T2–T3 had a higher mean age compared to patients in stage Ta–T1. Moreover, patients with high-grade papillary urothelial carcinoma had a higher mean age compared to patients with urothelial papilloma.

Based on the nucleotide sequence analysis of the VP1 region, the BKPyV genotypes I and IV and the JCPyV genotypes 2, 3 and 6 were found among patients with bladder cancer in this study. Currently, 4 BKPyV genotypes (I, II, III, and IV) with 4 subtypes related to genotype I (Ia, Ib-1, Ib-2 and Ic) and six subtypes related to genotype IV (IVa-1, IVa-2, IVb-1, IVb-2, IVc-1 and IVc-2), and 8 JCPyV genotypes (1–8) with 5 subtypes related to genotype 2 (2a, 2b, 2c, 2d and 2e) have been identified depending on the geographical distribution [7, 26]. The BKPyV genotype I shows a widespread distribution and is prevalent in different parts of the world. The BKPyV genotype IV is predominant in Asia. The BKPyV genotypes II and III are less prevalent and are almost rare [27, 28]. The JCV genotypes 1 and 4 are predominant in the United States and Europe. The JCV genotypes 2 and 7 are prevalent in Asia. The JCV genotypes 3 and 6 are the main genotype in Africa. The JCV genotype 5 is a recombination of genotypes 2b and 6. The JCV genotype 8 has been isolated from Papua New Guinea and Western Pacific populations [26].

We have carried out the largest screening of BKPyV and JCPyV to date in an Iranian population with bladder cancer, finding that these viruses are unlikely to be effective causative factors in bladder carcinogenesis among patients with bladder cancer resident in the northern shores of the Persian Gulf. In addition, data on BKPyV and JCPyV infections in bladder cancer, including distribution by age, gender, place of residency, tumor grade and tumor stage, were analyzed. Besides, the high number of screened samples increases the generalizability of the results to the population resident in this region. In addition, this study highlighted the importance of testing multiple samples from different parts of the tumoral tissue for each patient. In this study, due to the limited number of non-cancerous bladder samples in the archives of the pathology department of the hospitals and difficulties in acquiring healthy bladder samples, only 30 non-cancerous samples were screened for detection of BKPyV and JCPyV. The low number of control patients is one of the limitations of this study. As another limitation, paraffin-embedded tissue samples were used to detect BKPyV and JCPyV. The detection rate of viral DNA might be slightly higher in fresh frozen tissue samples compared to paraffin-embedded tissue samples.

## Conclusion

According to the results of the present study, despite identifying BKPyV and JCPyV in a number of bladder cancer biopsy specimens and the high prevalence of bladder cancer among people resident in the South of Iran, it is suggested that these viruses are unlikely to be effective causative factors in bladder carcinogenesis in this region. However, alternative molecular techniques such as RNA-ISH, FISH, and IHC should be used in order to exclude the potential role of these two viruses in promoting bladder cancer. Moreover, our results showed no significant association between the prevalence of BKPyV and JCPyV and age, gender, place of residency, tumor grade and tumor stage. Therefore, environmental risk factors and genetic backgrounds may have a more prominent role than human polyomaviruses in the development of bladder cancer. Nevertheless, further studies are required to investigate these factors and their associations with bladder carcinoma in the South of Iran.

## Data Availability

All relevant data are within the paper.
